# Haemophilus influenzae invasive disease in the United States, 1994-1995: near disappearance of a vaccine-preventable childhood disease.

**DOI:** 10.3201/eid0402.980210

**Published:** 1998

**Authors:** K M Bisgard, A Kao, J Leake, P M Strebel, B A Perkins, M Wharton

**Affiliations:** Centers for Disease Control and Prevention, Atlanta, Georgia 30333, USA. kmb6@cdc.gov

## Abstract

We analyzed national Haemophilus influenzae (Hi) surveillance data from 1994 and 1995 to describe the epidemiology of Hi invasive disease among persons of all ages. Serotype data were available for 376 (56%) of 669 reported Hi cases among children aged 4 years or younger; 184 (49%) were H. influenzae type b (Hib). Among children aged 4 or younger, incidence (per 100,000) of all Hi invasive disease was 1.8 in 1994 and 1.6 (p < 0.05) in 1995. Children aged 5 months or younger had the highest average annual incidence rate of Hib invasive disease (2.2 per 100,000); children aged 6 to 11 months had the next highest rate (1.2 per 100,000)(p < 0.05). Of 181 children with Hib invasive disease whose age in months was known, 85 (47%) were too young (aged 5 months or younger) to have completed a primary series with an Hib-containing vaccine. Of the 83 children with known vaccination status who were eligible to receive a primary series (aged 6 months or older), 52 (63%) were undervaccinated, and the remaining 31 (37%) had completed a primary series in which vaccine failed. Among persons aged 5 years or older with Hi invasive disease, the lowest average annual incidence was among those 20 to 39 years of age (0.15 per 100,000), and the highest was among those aged 80 years or older (2.26 per 100,000). Among persons aged 5 years or older, serotype data were available for 1,372 (71%) of the 1,940 Hi invasive disease cases; 159 (28%) of the 568 Hi cases with known serotype were due to Hib.


### *Haemophilus influenzae* Invasive Disease in the United States, 1994–1995: Near Disappearance of a Vaccine-Preventable Childhood Disease

**Kristine M. Bisgard, Annie Kao, John Leake, Peter M. Strebel, Bradley A. Perkins, and Melinda Wharton**
Centers for Disease Control and Prevention, Atlanta, Georgia, USA

We analyzed national *Haemophilus influenzae* (Hi) surveillance data from 1994 and 1995 to describe the epidemiology of Hi invasive disease among persons of all ages. Serotype data were available for 376 (56%) of 669 reported Hi cases among children aged 4 years or younger; 184 (49%) were *H. influenzae* type b (Hib). Among children aged 4 or younger, incidence (per 100,000) of all Hi invasive disease was 1.8 in 1994 and 1.6 ($p < 0.05$) in 1995. Children aged 5 months or younger had the highest average annual incidence rate of Hib invasive disease (2.2 per 100,000); children aged 6 to 11 months had the next highest rate (1.2 per 100,000) ($p < 0.05$). Of 181 children with Hib invasive disease whose age in months was known, 85 (47%) were too young (aged 5 months or younger) to have completed a primary series with an Hib-containing vaccine. Of the 83 children with known vaccination status who were eligible to receive a primary series (aged 6 months or older), 52 (63%) were undervaccinated, and the remaining 31 (37%) had completed a primary series in which vaccine failed. Among persons aged 5 years or older with Hi invasive disease, the lowest average annual incidence was among those 20 to 39 years of age (0.15 per 100,000), and the highest was among those aged 80 years or older (2.26 per 100,000). Among persons aged 5 years or older, serotype data were available for 1,372 (71%) of the 1,940 Hi invasive disease cases; 159 (28%) of the 568 Hi cases with known serotype were due to Hib.

Before the first *Haemophilus influenzae* type b (Hib) polysaccharide vaccine was introduced in 1985, Hib was the most common cause of bacterial meningitis in children under 5 years of age (approximately 12,000 cases per year, most in children younger than 18 months [1]); approximately 5% of affected children died. Neurologic sequelae developed in 15% to 30% of the surviving children (1). An additional estimated 7,500 cases of other invasive Hib infections also occurred annually in young children (1). The cumulative risk for Hib invasive disease before the age of 5 was one in 200 children, similar to the risk for poliomyelitis during the 1950s (1,2).

Address for correspondence: Kristine M. Bisgard, Centers for Disease Control and Prevention, 1600 Clifton Road, Mail Stop E61, Atlanta, GA 30333, USA; fax: 404-639-8616; e-mail: kmb6@cdc.gov.

The incidence of Hib invasive disease among children aged 4 years or younger has declined by 98% since the introduction of Hib conjugate vaccines, initially licensed in 1989 for use in children aged 15 months or older, then for infants beginning at 2 months of age in 1990 (3-6). One goal of the Childhood Immunization Initiative (7) was to eliminate invasive Hib disease among children aged 4 years or younger by 1996; however, approximately 300 cases of *H. influenzae* (Hi) invasive disease per year continue to be reported (3).

Moreover, Hi invasive disease is an important cause of illness and death in immunocompromised persons and adults with certain underlying medical conditions (8-11). The average incidence of Hi invasive disease from 1988 to 1990 was estimated at 1.7 cases per 100,000; 50% of cases examined in this study were due to Hib (8).

Prior studies of Hi invasive disease among adults found case-fatality rates of 26% to 36% (8-11).

In this article, we summarize the epidemiology of Hi and Hib invasive disease among children aged 4 years or younger and persons aged 5 years or older in the United States during 1994 and 1995.

### Data Collection

We used three sources of surveillance data to document the number of reported Hi invasive disease cases with onset in 1994 and 1995. Cases were reported to the Centers for Disease Control and Prevention (CDC). Two sources were passive surveillance systems: the National Notifiable Diseases Surveillance System (NNDSS) and the National Bacterial Meningitis and Bacteremia Reporting System (NBMBRS); the third source was the active, laboratory-based surveillance sites.

The NNDSS case reports of Hi invasive disease are transmitted electronically from state health departments to CDC as part of the weekly notifiable disease reporting system. These reports contain only demographic information. Supplemental information, including Hi serotype, infection site, outcome, and Hib vaccination status and specific vaccine received among children aged 4 years or younger is transmitted electronically or mailed to CDC on NBMBRS reporting forms. During 1994, 10.5 million persons were under active, laboratory-based surveillance for Hi invasive disease: these included residents of the state of Oklahoma; three counties in the San Francisco Bay area of California; eight counties in the metropolitan area of Atlanta, Georgia; and four counties in Tennessee (3). In 1995, 12.8 million persons were under surveillance; an additional county in Tennessee was added, and Maryland was included in place of Oklahoma. Each site was contacted every 2 weeks to identify Hi invasive disease cases; surveillance personnel then collected detailed data on these cases. Data on cases from the active surveillance sites were also mailed to CDC on a form similar to the NBMBRS report form.

Information from the three data sources was combined, and duplicates (cases with identical date of birth, onset, county of residence, and demographic data) were eliminated. After the end of each calendar year, each state health department was contacted by telephone to send information on Hi invasive disease cases among children aged 4 years or younger not previously reported to CDC and to provide missing critical supplementary information, such as vaccinations received.

Our analysis included confirmed and probable cases. A confirmed case of Hi invasive disease was defined as an illness clinically compatible with invasive disease (such as meningitis, pneumonia, cellulitis, epiglottitis, peritonitis, pericarditis, septic arthritis, empyema, and abscesses) with isolation of Hi from a normally sterile site (e.g., cerebrospinal fluid or blood). A probable case was defined as an illness clinically compatible with detection of Hib antigen in cerebrospinal fluid (12). Up to three infection sites could be recorded from a list including primary bacteremia, meningitis, pneumonia, cellulitis, epiglottitis, peritonitis, pericarditis, septic arthritis, or another specified site.

Completion of a primary vaccination series was defined according to recommendations from the Advisory Committee on Immunization Practices (4). Hib vaccine doses administered fewer than 14 days before the onset of disease were excluded.

### Data Analysis

U.S. census population estimates for 1994 and 1995 were used to calculate age- and race-specific average annual incidence rates (calculated by [events/2] ÷ [population/2]) for all Hi invasive disease and for documented Hib disease (13). The difference between two rates was regarded statistically significant at the 0.05 level if it exceeded the following: $2 \times (R1^2/N1 + R2^2/N2)^{1/2}$, where R1 is the rate corresponding to N1 events and R2 is the rate corresponding to N2 events (14). County average annual incidence rates of Hi invasive disease were calculated by using 1990 county population estimates and were mapped with Atlas Mapmaker (15). For Hi invasive disease cases among children aged 4 years or younger, we looked at the proportion due to Hib, the Hib vaccination history, and the age-specific and geographic distribution of residual Hib cases. A chi-square test of independence was used to test the association between age category and outcome.

### Findings

In 1994 and 1995, 1,277 and 1,332 cases of invasive Hi disease were reported, respectively. Although the overall incidence of invasive Hi disease remained constant, the incidence of invasive Hi disease among children aged 4 years or younger was lower in 1995 (305 cases; 1.56 per 100,000) than in 1994 (364 cases; 1.84 per

100,000) ($p < 0.05$). During 1994 and 1995, children 5 months or younger had the highest reported disease incidence of Hi invasive disease (8.02 per 100,000 children), Hib invasive disease (2.20 per 100,000), and Hi invasive disease of unknown serotype (3.57 per 100,000) (Table 1). The incidence of Hi invasive disease, Hib invasive disease, and Hi invasive disease of unknown serotype was significantly greater ($p < 0.05$) among children aged 5 months or younger than among children 6 to 11 months of age, who had the next highest rates; a high rate of Hi invasive disease was also observed among adults aged 80 years or older. The lowest disease rates were found in persons 5 to 19 and 20 to 39 years of age.

Hi isolates were serotyped for only 36% of 2,609 reported cases. A higher percentage of isolates were serotyped among children aged 4 years or younger (56%) than among older persons (29%) (Table 2). Among Hi cases in children aged 4 years or younger with reported serotype information, Hib was the most common single serotype (184 type b of 376 serotyped isolates); Hib represented 49% of invasive disease and 65% of meningitis. The average annual incidence for Hib invasive disease was 0.47 per 100,000 children aged 4 years or younger. Among persons aged 5 years or older, Hib accounted for 28% of serotyped isolates.

Among the 433 Hi case-patients younger than 1 year of age for whom age in months was reported during the study period, most (72%) were aged 5 months or younger; 29% were younger than 1 month of age (Figure 1). Similarly, of 184 children aged 4 years or younger with Hib invasive disease, most (133, 72%) cases were in children younger than 1 year of age.

The highest average annual incidence rates (5.1 per 100,000) of Hi invasive disease among case-patients aged 4 years or younger were in American Indians; comparisons with other race or ethnic groups were statistically significant ($p < 0.05$) (Table 3). Similar race and ethnic differences were noted for Hib invasive disease incidence but not for Hi invasive disease of unknown serotypes. Among case-patients aged 5 years or older, differences between race or ethnic groups were less marked. The incidence rates among male and female case-patients aged 5 years or older and among case-patients aged 4 years or younger were equivalent.

Infection site data were available for 1,556 (60%) case-patients (Table 4). Bacteremia was the most frequently reported site of Hi invasive disease for both children aged 4 years or younger (62%) and persons aged 5 years or older (51%). The second most frequently reported site of

Table 2. Serotype information for reported Hi invasive disease (n=2609) and meningitis cases (n=380), stratified by age, United States, 1994–1995

| | No. (%) of children aged 0–4 years | | No. (%) of persons aged ≥ 5 years | |
|---|---|---|---|---|
| Serotype | Invasive disease | Meningitis | Invasive disease | Meningitis |
| Serotype b | 184 (27) | 101 (47) | 159 (8) | 41 (25) |
| Other[a] | 114 (17) | 39 (18) | 171 (9) | 26 (15) |
| Non-typeable | 78 (12) | 16 (8) | 238 (12) | 25 (15) |
| Unknown | 293 (44) | 57 (27) | 1,372 (71) | 75 (45) |
| Total | 669 (100) | 213 (100) | 1,940 (100) | 167 (100) |

[a]Includes serotypes a, c, d, e, f.

Table 1. Number of cases and average annual incidence rates of Hi and Hib invasive disease and Hi invasive disease of unknown serotype, by age,[a] United States, 1994–1995

| | Hi | | Hib | | Hi serotype unknown | |
|---|---|---|---|---|---|---|
| Age group | No.[b] | Incid.[c] | No.[b] | Incid.[c] | No.[b] | Incid.[c] |
| 0-5 mo | 310 | 8.02 | 85 | 2.20 | 138 | 3.57 |
| 6-11 mo | 123 | 3.18 | 45 | 1.16 | 45 | 0.16 |
| 1-4 yr | 219 | 0.69 | 51 | 0.16 | 99 | 0.31 |
| 5-19 yr | 179 | 0.16 | 23 | 0.02 | 108 | 0.10 |
| 20-39 yr | 249 | 0.15 | 25 | 0.02 | 166 | 0.10 |
| 40-59 yr | 405 | 0.33 | 42 | 0.03 | 280 | 0.23 |
| 60-79 yr | 715 | 1.01 | 51 | 0.07 | 539 | 0.76 |
| 80 + yr | 361 | 2.26 | 18 | 0.11 | 152 | 0.95 |

[a]Does not include 31 cases missing age and 17 patients aged <1 year but missing age in months.
[b]Number of cases over the 2-year period.
[c]Incidence per 100,000 population.

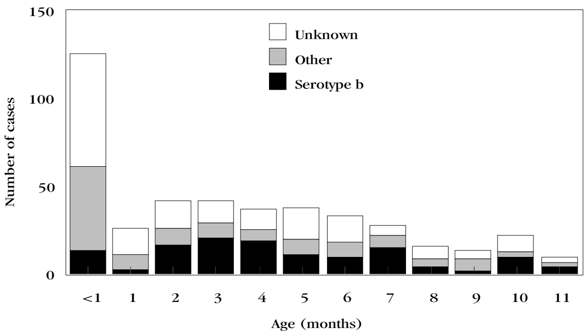

Figure 1. Reported Hi invasive disease cases by age and serotype, children aged <1 year,[a] United States, 1994–1995.

[a]N=433; excluding 17 children aged <1 year missing age in months.

Table 3. Number of cases and average annual incidence rates of Hi and Hib invasive disease and Hi invasive disease of unknown serotype, by race and ethnicity,[a] United States, 1994–1995

| | Children aged 0–4 years | | | | | | Persons aged ≥5 years | |
|---|---|---|---|---|---|---|---|---|
| | All Hi | | Hib | | Hi: unknown serotypes | | All Hi | |
| Race/ethnicity | No.[b] | Incidence[c] | No.[b] | Incidence[c] | No.[b] | Incidence[c] | No.[b] | Incidence[c] |
| Non-Hispanic | | | | | | | | |
| White | 338 | 1.34 | 102 | 0.40 | 150 | 0.59 | 1,269 | 0.35 |
| Black | 122 | 2.08 | 32 | 0.55 | 52 | 0.89 | 258 | 0.45 |
| American Indian | 18 | 5.10 | 10 | 2.83 | 3 | 0.84 | 12 | 0.37 |
| Asian or Pacific Islander | 13 | 0.86 | 3 | 0.20 | 6 | 0.40 | 21 | 0.13 |
| Hispanic | 94 | 1.49 | 7 | 0.11 | 31 | 0.49 | 70 | 0.15 |

[a]Does not include 84 (13%) of children aged ≤ 4 years, and 310 (16%) of persons aged ≥ 5 years for whom race and ethnicity data were missing.
[b]Number of cases over the 2-year period.
[c]Per 100,000 population.

invasive disease among children aged 4 years or younger was meningitis (44%), and among persons aged 5 years or older it was pneumonia (36%).

Outcome data were available for 1,380 (53%) of 2,609 case-patients with Hi invasive disease. Among case-patients with known outcome, the overall case-fatality ratio (CFR) was 14%. The CFR differed by age; the CFRs among case-patients aged 0 to 1 year, 1 to 29 years, 30 to 59 years, and 60 years of age or older were 10%, 6%, 13%, and 24%, respectively ($p < 0.001$).

During 1994 and 1995, a moderate seasonal pattern of Hi and Hib invasive disease was observed, with more cases occurring during the winter months (Figure 2); this pattern was similar for children 4 years of age or younger (data not shown).

To determine the remaining cases of Hib invasive disease, data on the number of Hib vaccine doses received were reviewed for the 184 reported cases among children aged 4 years or younger by the age (in months) of the case-patient (Table 5). Eighty-five (47%) of the 181 children aged 4 years or younger with known age in months were too young (5 months or younger) to have completed a primary series with the most commonly used Hib vaccines (three-dose primary series). Of the 149 children aged 4 years or younger with known vaccination status, 83 were eligible (aged 6 months or older) to receive a primary series; of these, 52 (63%) were undervaccinated. Thirty-one (37%) of the 83 children had completed a primary series with either three doses of HbOC (Wyeth-Lederle Laboratories, Pearl River, NY) or PRP-T (Pasteur Mérieux Connaught, Lyon, France), or with two doses of PRP-OMP (Merck, Inc., West Point, PA), or one dose with any Hib-containing vaccine on or after 15 months of age. Manufacturer and vaccine lot information was available for 22 of the

Table 4. Reported infection site[a] among Hi invasive disease cases,[b] United States, 1994–1995

| | Number (%) | |
|---|---|---|
| Site | Children aged 0-4years[c] | Persons aged ≥5 years[d] |
| Bacteremia[e] | 299 (62) | 551 (51) |
| Meningitis | 213 (44) | 167 (16) |
| Pneumonia | 42 (9) | 390 (36) |
| Cellulitis | 19 (4) | 7 (1) |
| Epiglottitis | 8 (2) | 19 ( 2) |

[a]Includes case-patients with multiple infection sites, such as meningitis and bacteremia.
[b]Case-patients with known infection site (n=1,556).
[c]N=485.
[d]N=1,071.
[e]Bacteremia is reported following positive blood culture results, with or without foci.

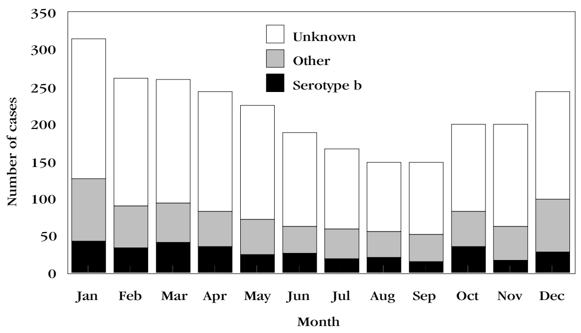

Figure 2. Reported Hi invasive disease cases by serotype and month of onset,[a] United States, 1994–1995.
[a]N=2,609; excluding 10 cases with unknown month of onset.

Table 5. Children aged ≤4 years with invasive Hib disease,[a] by number of Hib vaccine doses received and age group, United States, 1994–1995

| Age (mos.) | Rec.[c] | No. of children receiving vaccine[b] 0 | 1 | 2 | 3 | 4 | Vaccination status unknown | Total |
|---|---|---|---|---|---|---|---|---|
| 0-1 | 0 | 15 | | | | | 2 | 17 |
| 2-3 | 1 | 17 | 12 | | | | 9 | 38 |
| 4-5 | 2 | 7 | 13 | 2 | | | 8 | 30 |
| 6-11 | 3 | 13 | 8 | 11 | 6 | | 7 | 45 |
| 12-18 | 4 | 5 | 1 | 2 | 7 | 2 | 0 | 17 |
| 19-59 | 4 | 11 | 5 | 1 | 7 | 4 | 6 | 34 |
| Total | 4 | 68 | 39 | 16 | 20 | 6 | 32 | 181 |

[a]Excludes three children aged <1 year but missing age in months; vaccination status was unknown for two of the children, and one child was unvaccinated.
[b]Five children completed a primary series with 2 doses of PedvaxHIB (at ages 7, 11, 13, 14, and 43 months); two children completed a primary series with 1 dose at ≥15 months of age.
[c]Cumulative recommended doses for PRP-T and HbOC vaccines.

31 children who had received a primary series. No single lot predominated, and no child received vaccine from the same lot for all doses.

Overall, 49 states and 673 (21%) of the 3,137 U.S. counties reported at least one Hi invasive disease case with disease onset during the 2-year period; of these, 327 (49%) counties (from 47 states) reported two or more Hi cases (Figure 3A). Forty-seven states and 339 (11%) of counties reported Hi invasive disease cases among children aged 4 years or younger, and 42 states and 137 (4%) counties reported Hib cases during the 2-year period; 25 (18%) of these counties reported two or more Hib cases (range 2 to 8 cases). In these 25 counties, the incidence for Hib invasive disease was 0.41 to 12.19 per 100,000 children aged 4 years or younger (Figure 3B). Among the 14 counties with two Hib cases, the median time between cases was 139 days (range 14 to 334 days). In three of the 11 counties with three or more Hib invasive disease cases, children were too young (aged 5 months or younger) to have completed a primary series with an Hib vaccine; in the remaining eight counties, 20 (67%) of 30 children had been eligible for vaccination (aged 6 months or older) and only one child had completed a primary series. In these 11 counties, the incidence for Hib invasive disease was 0.41 to 4.31 per 100,000 children aged 4 years or younger.

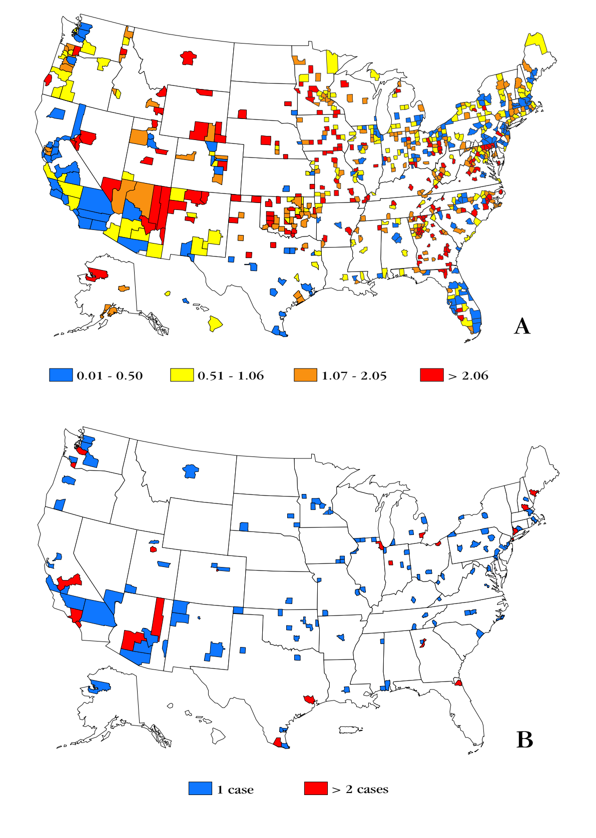

Figure 3. A. Average annual incidence of Hi invasive disease among persons of all ages, by county, United States, 1994–1995. B. Reported number of Hib invasive disease cases among children aged ≤ 4 years, by county, United States, 1994–1995.

### Conclusions

Invasive Hib disease among children aged 4 years or younger in the United States has become rare because of the introduction and widespread use of conjugate Hib vaccines. Although national vaccination coverage of children 19 to 35 months of age with three or more doses of Hib conjugate vaccines only reached 90% in 1995 and some metropolitan areas had lower coverage rates (16,17), all Hi invasive disease incidence in 1994 and 1995 was 98% lower than during the prevaccine era. During that era, Hib invasive disease was the cause of more than 95% of Hi invasive disease (18-22). While our results were not directly comparable to prevaccine Hib invasive disease estimates because of reliance on passive reporting and lack of serotype data (23), in the active surveillance sites, the race-adjusted incidence of Hib invasive disease declined 99% from 1989 to 1995 (3). Before Hib conjugate vaccine availability in 1988, the highest Hib invasive disease incidence had been in children 6 to 17 months of age (estimated annual incidence of 275 and 223 per 100,000 for children 6 to 11 and 12 to 17 months of age, respectively), compared with children 5 months of age or younger (estimated annual incidence of 148 per 100,000) (18-22). However, during 1994 and 1995, children aged 5 months or younger had the highest incidence of Hib invasive disease of all age groups (2.2 per 100,000) (Table 1); these children were too young to have completed a primary vaccination series.

Consistent with previous studies of Hib invasive disease in children aged 4 years or younger in the prevaccine era, we found higher Hi and Hib disease incidence among American Indian (the highest incidence rates), black, and Hispanic children than among Asian or Pacific islander and white children (18,20). In the prevaccine era, Hib invasive disease rates among American Indian children were approximately five times higher than among non-American Indian children in Alaska (annual incidence of 601 per 100,000 children) (20). Although important progress has been made in reducing Hib invasive disease in this population, American Indian children continue to have higher disease incidence than other racial or ethnic groups. The reasons for the higher Hib invasive disease incidence among some ethnic or racial groups or some regions are unknown but could be associated with one or more of the following: low Hib vaccine coverage levels in an area (e.g., an inner-city “pocket of need”), low socioeconomic status, cofactors, host susceptibility, and high levels of carriage in the population. In 1996, a study of Alaskan native children aged 6 years or younger with high vaccination coverage (>90%) found a carriage level of 8%, a rate similar to that in the prevaccine era (24). However, a 1992 study in the Atlanta, Georgia, metropolitan area documented a low (0.2%) carriage level among children with a 75% Hib vaccination coverage (25). In the United States, the reduction in the number of reported cases has been greater than expected when the efficacy of Hib conjugate vaccines and the coverage of a primary series alone are taken into account; this is likely due to the reduction of pharyngeal carriage in vaccinated children, which protects nonvaccinated persons in the community (i.e., herd immunity) (18,26). High carriage levels are unlikely in most areas of the United States; the difference in the results of the two studies (24,25) may be due to the use of different Hib conjugate vaccines, as well as to different host and environmental factors. In European countries that use Hib conjugate vaccines, increasing vaccine use has been associated with diminishing rates of both Hib pharyngeal carriage and invasive Hib disease (26).

With the marked decline among children, the occurrence of Hib invasive disease among older persons (aged 5 years or older) has achieved greater prominence. Although serotype data were missing from most cases, reported cases among both children (n = 184) and adults (n = 159) indicate persistent circulation of Hib. Reported Hib invasive disease cases and nasopharyngeal carriage among children have decreased; however, because these data were from passive reporting sources it is not clear if a similar decrease in invasive Hib disease in older children and adults has occurred nationally. Because of underreporting of adult Hib invasive disease cases nationally, 1994 to 1995 incidence rates cannot be directly compared with rates found in prior studies (8-11). In our study, 28% of serotyped isolates were Hib; however, among adults in the Atlanta, Georgia, metropolitan active surveillance site, Hi invasive disease cases due to Hib declined in 1990 to 1991 from 50% to 0% in 1995 to 1996 (8,27). Nevertheless, because previous studies have found that most Hi and Hib invasive disease occurred among immunocompromised patients or patients with underlying conditions (e.g., chronic lung disease, splenectomy, leukemia, HIV infection, sickle-cell disease), health-care providers should consider Hib vaccination for patients at high risk (6). Because of continued occurrence of Hib invasive disease among older persons and infants too young to be vaccinated, additional strategies may be needed to eliminate Hib invasive disease among children aged 4 years or younger. In addition, ongoing surveillance for Hi invasive disease is needed to monitor incidence among older persons; case investigations may include assessments for underlying immunosuppressive conditions or ascertainment of other cofactors such as prior or concomitant viral infections.

Continued decline in incidence of Hib invasive disease in the United States requires that children aged 4 years or younger be appropriately vaccinated to protect them and other children and infants in the community. Intensive efforts to increase vaccination levels among children in areas with coverage rates lower than 90% are needed to reach the elimination goal. Children should receive a primary Hib vaccination series at ages 2, 4, and 6 months with either HbOC or PRP-T, or at ages 2 and 4 months with PRP-OMP (6). A booster dose with any of the four licensed Hib conjugate vaccines should be given at 12 to 15 months of age (6). Among children who were eligible for vaccination, timely completion of a primary series might have prevented 63% of reported Hib invasive disease cases in 1994 and 1995. Few (28) Hib cases among children 4 years or younger were reported in those who had completed a primary series with Hib vaccine, which suggests that vaccine failure was uncommon. However, because of passive reporting, vaccination status and vaccine failure may be underestimated. Hib invasive disease in children too young to have completed a primary series will continue to be a barrier for elimination unless Hib nasopharyngeal carriage rates in the population (including adults) approach zero.

Because the highest Hib invasive disease incidence occurs among infants, all infants should be appropriately vaccinated, beginning at 2 months of age. In a case-control study for undervaccination among Hib invasive disease cases among children aged 2 to 18 months in the United States, risk factors for undervaccination included low maternal education and having a single mother (29); special outreach efforts may be needed to vaccinate children at risk. Other programmatic strategies to increase vaccination rates include linkage of the Special Supplemental Nutrition Program for Women, Infants and Children (WIC) to vaccination assessment and referral (30), implementation of immunization registries and reminder/recall systems to encourage parents to vaccinate their children in a timely fashion (28,31,32), and monitoring clinic coverage levels and providing feedback to clinic staff (33). In addition to high vaccination coverage of other children in the community to help reduce carriage, breast-feeding of infants should be promoted because it is protective against invasive Hi disease in this age group (21). For children aged 4 years or younger with Hib invasive disease who have completed a primary Hib vaccination series, case investigations may include examining reasons for vaccine failure, such as measuring antibodies to the polyribosylribitol phosphate (PRP) capsule of Hib; undetectable levels suggest lack of immune response (18).

To monitor the changing epidemiology of Hib invasive disease and look for an increase or decline in incidence among children and adults, serotype information for all Hi invasive disease cases is essential and will generate data needed to examine new strategies for elimination. Surveillance is necessary for detecting pockets of continuing transmission and identifying areas in need of improved vaccine coverage (3). Each case of Hib invasive disease is a sentinel for circulation of Hib in a community. In addition, Hib invasive disease cases among children aged 4 years or younger may be a marker for low community coverage. Therefore, community immunization policies, practices, and coverage levels should be reviewed, and recommendations should be made, if needed, to improve Hib vaccination coverage levels.

### Acknowledgments

The authors thank Wendy Wattigney for her review of the manuscript and assistance with statistical analysis and Shalani Desai for her meticulous assistance in data cleaning.

Dr. Bisgard is a medical epidemiologist in CDC's National Immunization Program. Her research interests include vaccine effectiveness, uses of new vaccines, and outbreak control of vaccine-preventable diseases; her areas of expertise include vaccine preventable diseases (Hib, pertussis, and diphtheria) and infectious disease epidemiology.

### References

1. Cochi SL, Broome CV, Hightower AW. Immunization of U.S. children with *Haemophilus influenzae* type b polysaccharide vaccine: a cost-effectiveness model of strategy assessment. JAMA 1985;253:521-9.
2. Strebel PM, Sutter RW, Cochi SL, Biellik RJ, Brink EW, Kew OM, et al. Epidemiology of poliomyelitis in the United States one decade after the last reported case of indigenous wild virus-associated disease. Clin Infect Dis 1992;14:568-79.
3. Centers for Disease Control and Prevention. Progress towards elimination of *Haemophilus influenzae* type b disease among infants and children—United States, 1987-1995. MMWR Morb Mortal Wkly Rep 1996;45:901-6.
4. Adams WG, Deaver KA, Cochi SL, Plikaytis BE, Bell ER, Broome CV, et al. Decline of childhood *Haemophilus influenzae* type b (Hib) disease in the Hib vaccine era. JAMA 1993;269:221-6.

5. Centers for Disease Control. *Haemophilus* b conjugate vaccines for prevention of *Haemophilus influenzae* type b disease among infants and children two months of age and older: recommendations of the Immunization Practices Advisory Committee (ACIP). MMWR Morb Mortal Wkly Rep 1991;40(RR-1):1-6.
6. Centers for Disease Control and Prevention. Recommendations for use of *Haemophilus* b conjugate vaccines and a combined diphtheria, tetanus, pertussis and *Haemophilus* type b vaccine: Recommendations of the Immunization Practices Advisory Committee (ACIP). MMWR Morb Mortal Wkly Rep 1993;42(RR-13):1-15.
7. Centers for Disease Control and Prevention. Reported vaccine-preventable disease—United States, 1993, and the Childhood Immunization Initiative. MMWR Morb Mortal Wkly Rep 1994;43:57-60.
8. Farley MM, Stephen DS, Brachman PS, Harvey C, Smith JD, Wenger JD, et al. Invasive *Haemophilus influenzae* disease in adults. A prospective, population-based surveillance. Ann Intern Med 1992;116:806-12.
9. Takala AK, Eskola J, van Alpen L. Spectrum of invasive *Haemophilus influenzae* type b disease in adults. Arch Intern Med 1990;150:2573-6.
10. Steinhart R, Reignold AL, Taylor F, Anderson G, Wenger JD. Invasive *Haemophilus influenzae* infections in men with HIV infection. JAMA 1992;268:3350-2.
11. Kostman JR, Sherry BL, Filgner CL, Egaas S, Sheeran P, Baken L, et al. Invasive *Haemophilus influenzae* infections in older children and adults in Seattle. Clin Infect Dis 1993;17:389-96.
12. Centers for Disease Control and Prevention. Case definitions for infectious conditions under public health surveillance. MMWR Morb Mortal Wkly Rep 1997;46(RR-10):15.
13. Resident population—estimates by age, sex, and Hispanic origin. Washington: U.S. Dept. of Commerce, Bureau of the Census, Population Division; 1995 July. Appendix A.
14. Technical notes: nature and sources of data. Hyattsville (MD): Centers for Disease Control and Prevention, National Center for Health Statistics; 1992. Monthly vital statistics report 41:21.
15. Atlas GIS Software (computer program). Arlington (VA): ATLAS Software, Claritas, Inc.; **(Au: give date).**
16. Centers for Disease Control and Prevention. Vaccination coverage levels of 2-year-old children—United States, January-March 1994. MMWR Morb Mortal Wkly Rep 1995;44:142-50.
17. Centers for Disease Control and Prevention. National, state, and urban area vaccination coverage levels among children aged 19-35 months—United States, January-December 1995. MMWR Morb Mortal Wkly Rep 1997;46:176-82.
18. Ward J, Leiberman JM, Cochi SL. *Haemophilus influenzae* vaccines. Vaccines. 2nd ed. In: Plotkin SA, Mortimer EA, editors. Philadelphia: WB Saunders Co. 1994;337-86.
19. Murphy TV, Osterholm MT, Pierson LM, White KE, Breedlove LVN, Siebert GB, et al. Prospective surveillance of *Haemophilus influenzae* type b disease in Dallas County, Texas and in Minnesota. Pediatr 1987;79:173-80.
20. Ward JI, Lum MKW, Hall DB, Silimperi DR, Bender TR. Invasive *Haemophilus influenzae* type b disease in Alaska: background epidemiology for a vaccine efficacy trial. J Infect Dis 1986;153:17-26.
21. Cochi SL, Fleming DW, Hightower AW, Limpakarnjanarat K, Facklam RR, Smith JD, et al. Primary invasive *Haemophilus influenzae* type b disease: a population-based assessment of risk factors. J Pediatr 1986;108:887-96.
22. Granoff DM, Basdon M. *Haemophilus influenzae* infections in Fresno County, California: a prospective study of the effects of age, race, and contact with a case on incidence of disease. J Infect Dis 1980;141:40-6.
23. Standaert SM, Lefkowitz LB, Horan JM, Hutcheson RH, Schaffner W. The reporting of communicable diseases: a controlled study of *Neisseria meningitidis* and *Haemophilus influenzae* infections. Clin Infect Dis 1995;20:30-6.
24. Galil K, Singleton R, Levine O, Fitzgerald M, Ajello G, Bulkow L, Parkinson A. High prevalence of *Haemophilus influenzae* type b (Hib) carriage among Alaska Natives despite widespread use of Hib conjugate vaccine. In: Abstracts of the 35th Infectious Disease Society of America; San Francisco, California; 1997 Sep 13-16; Abstract 421. Alexandria (VA): Infectious Disease Society of America; 1997.
25. Mohle-Boetani JC, Ajello G, Breneman E, Deaver KA, Harvey C, Plikaytis BD, et al. Carriage of *Haemophilus influenzae* type b in children after widespread vaccination with conjugate *Haemophilus influenzae* type b vaccines. Pediatr Infect Dis J 1993;12:589-93.
26. Barbour ML. Conjugate vaccines and the carriage of *Haemophilus influenzae* type b. Emerg Infect Dis 1996;3:176-82.
27. Farley MM, Baughman W, Robinson K, Facklam R, Schuchat A, Wenger JD. Elimination of invasive *Haemophilus influenzae* type b (Hib) disease in adults in Atlanta through immunization of children with conjugate Hib vaccine. In: Abstracts of the 37th Interscience Conference on Antimicrobial Agents and Chemotherapy; Toronto, Canada. Abstract K-150. Washington: American Society for Microbiology, Washington; 1997. p. 352.
28. Jafari H, Adams W, Deaver K, Plikaytis B, Wenger J. Efficacy of *Haemophilus influenzae* Type b (Hib) conjugate vaccine, risk factors for invasive Hib disease and under-vaccination in the United States. Abstracts of the Interscience Conference on Antimicrobial Agents and Chemotherapy; San Francisco, California; 1995; Abstract # G12. Washington: American Society for Microbiology; 1995. p. 160.
29. Centers for Disease Control and Prevention. Recommendations of the Advisory Committee on Immunization Practices: programmatic strategies to increase vaccination coverage by age 2 years—linkage of vaccination and WIC services. MMWR Morb Mortal Wkly Rep 1996;45:217-8.
30. Campbell JR, Szilagyi PG, Rodewald LE, Doane C, Roghmann KJ. Patient-specific reminder letters and pediatric well-child-care show rates. Clin Pediatr (Phila) 1994;May:268-72.

31. Abramson JS, O'Shea MO, Ratledge DL, Lawless MR, Givner LB. Development of a vaccine tracking system to improve the rate of age-appropriate primary immunization in children of lower socioeconomic status. J Pediatr 1995;126:583-6.
32. Dini EF, Linkins RW, Chaney M. Effectiveness of computer-generated telephone messages in increasing clinic visits. Arch Pediatr Adolesc Med 1995;149:902-5.
33. Centers for Disease Control and Prevention. Recommendations of the Advisory Committee on Immunization Practices: programmatic strategies to increase vaccination rates—assessment and feedback of provider-based vaccination coverage information. MMWR Morb Mortal Wkly Rep 1996;45:219-20.
